# Long-Term Self-Management of Vaginal Cube Pessaries Can Improve Sexual Life in Patients with Pelvic Organ Prolapse, Results from a Secondary Analysis

**DOI:** 10.1007/s00192-024-05882-5

**Published:** 2024-08-05

**Authors:** Zoltan Nemeth, Peter Vida, Predrag Markovic, Peter Gubas, Kalman Kovacs, Balint Farkas

**Affiliations:** 1 Department of Gynecology, Brothers of St. John of God Hospital Vienna, Vienna, Austria; 2 Department of Obstetrics and Gynecology, University of Pecs School of Medicine, 17 Edesanyak Str., Pecs, Hungary; 3 Department of Obstetrics and Gynecology, B-A-Z County Teaching Hospital, Miskolc, Hungary; 4 National Laboratory on Human Reproduction, University of Pécs, 17 Édesanyak Str., 7624 Pecs, Hungary; 5 HUN-REN-PTE Human Reproduction Research Group, 17 Édesanyak Str., 7624 Pecs, Hungary

**Keywords:** Vaginal pessary, Self-management, Pelvic floor disorders, Pelvic organ prolapse, Sexual life, Sexual wellbeing

## Abstract

**Introduction and Hypothesis:**

Currently, little is known about how daily self-management of cube pessaries influences sexual function. We hypothesized that removing the cube pessary prior to sexual activity did not negatively influence the sexual function, and pessary self-care did not lead to a deterioration of sexual wellbeing.

**Methods:**

We conducted a planned secondary analysis of a prospective cohort study in which 214 patients with symptomatic pelvic organ prolapse (stage 2+) were enrolled (2015). Each patient was size fitted with a cube pessary and completed a questionnaire online or by phone ≥ 5 years after her initial fitting. Changes in quality of life were measured using the Patient Global Impression of Improvement (PGI-I).

**Results:**

Of the 143 women included in our analyses, 92 (64.3%) were sexually active during the study period. These patients (73.9%; 68 out of 92) described their sexual wellbeing as “better” or “much better” than their pretreatment status. Sexually active patients had a better quality of life as measured by the PGI-I than the sexually inactive patients. Of the sexually active patients, 91.3% (84 out of 92) described their condition as “better” or “much better” than their pretreatment status, whereas 84.3% (43 out of 51) of the sexually inactive patients reported the same improvement. Over 90% of sexually active patients reported that removal of the vaginal cube pessary before sexual activity is not disruptive.

**Conclusions:**

The overwhelming majority of the patients with symptomatic pelvic organ prolapse using daily self-management of cube pessaries reported that removal of the vaginal cube pessary before sexual activity is not disruptive, and its use was accompanied by improved sexual wellbeing.

## Introduction

Pelvic organ prolapse (POP) is a condition involving loss of anatomical support of the pelvic organs that results in decreased quality of life (QoL) in symptomatic cases [1, 2]. Although the exact incidence of POP is unknown, the lifetime risk of undergoing surgery for POP in women has been estimated to range from 11 to 19%, indicating that the condition is relatively common [3–5].

Pelvic organ prolapse is often associated with sexual dysfunction [6], which can arise both from physical factors due to organ displacement and from psychological factors, including negative impacts on body image [2, 7]. POP-related sexual symptoms include abstinence, vaginal wind, vaginal laxity, obstructed intercourse, and dyspareunia [8]. These symptoms can lead to decreased sexual desire, arousal, and orgasm [9]. Although most literature describing sexual activity among pessary users means vaginal intercourse, it is important to consider that sexual activity and habits vary with age, especially in elderly patients [10].

Vaginal pessaries have been used to treat POP since ancient times and were first reported in 400 BC [11]. Modern pessaries are made of hypoallergenic silicone and can be categorized into two major types: space-filling and support pessaries. According to current guidelines, conservative, nonsurgical treatment should be the first line of POP therapy [12, 13]. Most women can be fitted successfully with a pessary, and pessary use has been associated with high satisfaction rates and low rates of minor complications [13]. Therefore, pessaries should be considered in the treatment of women presenting with symptomatic POP and/or stress urinary incontinence [6]. Indeed, nearly two thirds of women with symptomatic POP choose to proceed initially with conservative case management [14].

A recently published meta-analysis concluded that there is no deterioration and some evidence of an improvement in sexual function in sexually active women who successfully use a pessary to treat POP [15]. Most of the present data originate from studies of ring pessaries. In a recent study, we examined the use of cube pessaries and found them to be a safe and effective treatment for improving POP-related symptoms and QoL in the long term [16]. In the present study, we aimed to evaluate the long-term (5-year) self-reported sexual satisfaction rates in women using cube pessaries to treat symptomatic POP. We hypothesized that removing the cube pessary prior to sexual activity would not negatively influence sexual function. Second, we hypothesized that pessary self-care would not lead to a deterioration in sexual wellbeing.

## Materials and Methods

### Study Population

This study was a planned secondary analysis of a prospective cohort study (IV/7737–3/2021/EKU) that investigated the daily self-management of cube pessaries by patients with POP [15]. The target population was a group of women (*n* = 214) who began a conservative treatment plan for POP at least 5 years prior to collection of data for this study, which took place from May to August 2021. All participants provided informed consent. All recruited patients had their initial pessary fitting between January and December of 2015. At their initial fittings, the participants were examined according to the guidelines established by the International Urogynecological Association.

The inclusion criteria were symptomatic (bulge sensation in their vagina with or without symptoms of urinary, bowel, or sexual dysfunction), stage 2 or higher POP of the anterior, middle, and/or posterior compartments of the vagina, and successful fitting with a vaginal cube pessary (Dr. Arabin^®^) for daily self-management. The exclusion criteria were any active infection of the pelvis or vagina (e.g., vaginitis, pelvic inflammatory disease), physical or mental inability to manage independent use of the pessary, or discontinuation of pessary use for any reason within 4 weeks of the fitting.

Participants completed a questionnaire online (using a link sent via e-mail) or by telephone interview if they did not have access to the internet.

### Data Collection

The data were anonymized. For each patient, we recorded basic demographic data, including age and body mass index (BMI). Alterations in pelvic anatomy were classified according to the Pelvic Organ Prolapse Quantification system [17]. All terminology used followed the recommendations of the International Continence Society.

The sizes of the space-filling cube pessaries were designated 0–5, corresponding to the following respective diameters/volumes: size 0, 25 mm/15 cm^3^; size 1, 29 mm/24 cm^3^; size 2, 32 mm/30 cm^3^; size 3, 37 mm/42 cm^3^; size 4, 41 mm/60 cm^3^; and size 5, 45 mm/84 cm^3^ [18]. At the fitting stage, we emphasized that pessary therapy is like wearing eyeglasses in that they are medically assistive devices that can eliminate symptoms immediately during their use [19]. We informed the patients that the pessary is easy to use and cost-effective and that daily pessary use should not disrupt their sexual activity [19]. Appropriate pessary size was determined for each individual by selecting a pessary that was large enough to resolve the patient’s POP symptoms while being small enough to avoid discomfort [19]. The patients received detailed instructions on pessary use and care, and particular attention was given to the removal technique. Postmenopausal women were advised to apply local estrogen cream (estriol, Ovestin®) replacement therapy twice a week together with the therapy. Patients were asked to return for annual check-ups and at any other time if they had a complaint.

The questionnaire included questions about the participant’s sex life and any changes following pessary use and self-management. Changes in QoL following pessary use were assessed using the validated Patient Global Impression of Improvement (PGI-I) scale [20]. The treatment plan was considered successful at 5 years if the patient used the pessary regularly and wished to continue its use. Patients were considered lost to follow-up if they could not be contacted, either online or by telephone.

### Statistical Analysis

Statistical analyses were performed using SPSS version 20 (IBM, Armonk, NY, USA) at the University of Pecs Institute of Bioanalysis. Continuous data are presented as means with standard deviations (SDs), and categorical data are presented as the number of observations or percentage values. Fisher’s exact test was applied to independence analyses performed between categorical variables. Statistical significance was set at *p* < 0.05 or *p* < 0.1, as indicated.

## Results

### Demographics

Of the 214 patients who accessed the online questionnaire or were contacted by phone, 185 completed the questionnaire fully (86.45%). Of those 185 patients, 11 were excluded from this study because they did not have a successful initial fitting and they discontinued pessary use within 1 month. More than four-fifths of the study participants (81.2%; 143 out of 174) reported using a cube pessary successfully for at least 5 years after the initial fitting. Thus, data from these 143 patients were included in our secondary analysis of sexual function in women using daily self-management with pessaries. Of these patients, 92 (64.3%) were sexually active during the study period. All premenopausal (41 out of 41, 100%) and half of the postmenopausal patients (51 out of 102, 50%) were sexually active.

The mean (± SD) age of the participants was 61 ± 13 years (range 33–91 years), and the mean BMI was 25.99 ± 4.06 kg/m^2^ (range 15.63–43.28 kg/m^2^). The characteristics of the premenopausal and postmenopausal patients are compared in Table 1. The groups were similar with the exceptions that the postmenopausal group was older and had a higher mean BMI.

**Table 1:** Comparison of characteristics between premenopausal and postmenopausal patient groups

Characteristics of participants	Premenopausal (age ≤ 51), *n* = 41	Postmenopausal (age > 51), *n* = 51	*p* value
Demographic data			
Age (median, years) ± SD	47 ± 5.7	68 ± 8.0	< 0.001
BMI (kg/m^2^) ± SD	24 ± 4.6	27 ± 4.0	< 0.001
Delivery, median (minimum; maximum)	2 (0; 4)	2 (0; 5)	0.868
Obstetric data			
C-section rate (%)	19.0	6.03	0.031
Vacuum extraction rate (%)	10.3	5.17	0.205
Forceps delivery (%)	1.72	1.72	1.000
Gynecological data			
Abdominal hysterectomy rate (%)	0	11.2	0.023
Vaginal hysterectomy rate (%)	1.72	6.89	0.122
Colporrhaphy rate (%)	20.3	37.1	0.138
Other anti-POP operation rate (%)	0	2.40	0.554

Fisher’s exact test was applied to independence analyses performed between categorical variables

Statistical significance was set at *p* < 0.05 or *p* < 0.1 as indicated in the methods

### Sexual Activity and Changes in Sexual Wellbeing

All premenopausal (41 out of 41, 100%) and half of the postmenopausal patients (51 out of 102, 50%) reported that they were sexually active at the time they completed the questionnaire. The majority of premenopausal women described their sexual wellbeing as much better (7 out of 41, 17.0%) or better (20 out of 41, 48.8%) than before the use of the vaginal cube pessary, whereas 34.2% (14 out of 41) reported no change. More postmenopausal sexually active women reported an improvement in their sexual QoL, with 22.6% (11 out of 51) describing their sexual life as much better and 58.2% (30 out of 51) describing it as better. Only 19.6% of the postmenopausal women reported no change in their sexual life. No patients reported a deterioration in their sexual life due to the use of the pessary. The distribution of responses among sexually active pre- and postmenopausal women is presented in Table 2.

**Table 2:** Self-reported changes in sexual life in premenopausal and postmenopausal patients with pelvic organ prolapse after 5 years of cube pessary use

Changes in sexual wellbeing	Premenopausal *n* = 41), %	Postmenopausal (*n* = 51), %	*p* value
Much better	17.0	21.5	0.245
Slightly better	48.8	58.9	0.086
No change	34.2	19.6	0.043
Slightly worse	0	0	NA
Much worse	0	0	NA

Question: “How do you rate your sexual wellbeing compared to your pretreatment condition?”

Fisher’s exact test was applied to independence analyses performed between categorical variables

Statistical significance was set at *p* < 0.05 or *p* < 0.1, as indicated in the methods

### The Influence of Pessary Removal Prior to Vaginal Intercourse

Notably, most of the sexually active patients reported that removal of the vaginal cube pessary before sexual intercourse is convenient. The majority (67.4%) of respondents described pessary removal as “not disruptive,” and 25% rated pessary removal as “slightly disruptive.” Only 7.6% (7 out of 92) of patients reported that cube pessary removal was “disruptive” or “very disruptive” prior to intercourse (Table 3).

**Table 3:** Self-reported scores for pessary removal before sexual activity among long-term premenopausal and postmenopausal cube pessary users after 5 years of cube pessary use

Removing the pessary before sexual intercourse (rating)	Sexual life, % (*n*)		*p* value
	Premenopausal (41)	Postmenopausal (51)	
Not disturbing at all (1)	61 (25)	72.5 (37)	0.217
Barely disturbing (2)	29.3 (12)	19.6 (10)	0.123
Rather disturbing (3)	7.3 (3)	3.9 (2)	0.345
Very much disturbing (4)	2.4 (1)	2 (1)	NA

Question: “In your opinion, how much does the fact that you have to remove the device from the vagina before sexual intercourse affect your sexual life negatively?”

Fisher’s exact test was applied to independence analyses performed between categorical variables

Statistical significance was set at *p* < 0.05 or *p* < 0.1, as indicated in the methods

### Improvement in QoL After 5 Years of Daily Self-Therapy with a Cube Pessary

**Fig. 1:**
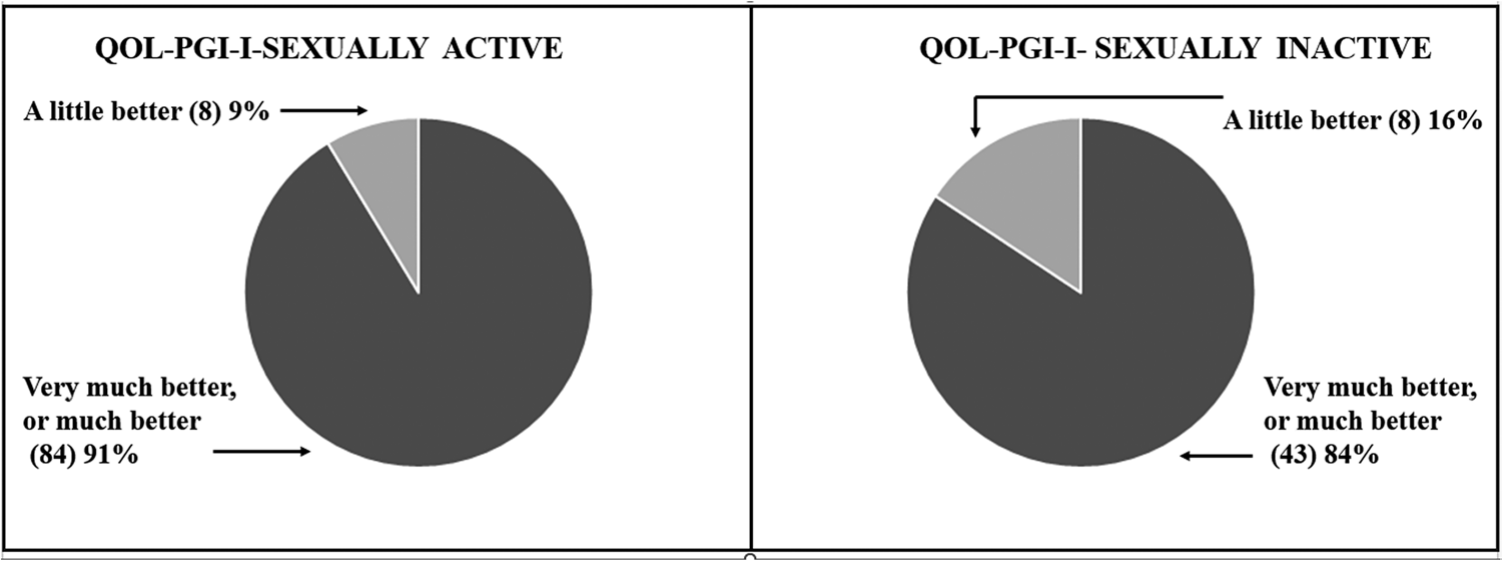
Quality of life (QoL) as measured by the Patient Global Impression of Improvement (PGI-I) after 5 years of daily self-therapy with cube pessaries in sexually active (*n* = 92) and sexually inactive (*n* = 51) patients

We used the PGI-I to assess the impact of daily vaginal cube pessary self-therapy on QoL. Comparing the data of sexually active and sexually inactive women, we found that the sexually active patients had a better overall QoL than the sexually inactive patients. Among the sexually active women, 91% (84 out of 92) described their QoL as “much better” or “better,” and 9% (8 out of 92) described their QoL as “slightly better.” In the sexually inactive participants, 84% (43 out of 51) reported their QoL as “much better” or “better” and 16% (8 out of 51) described their QoL as being “slightly better” (Fig. 1). The differences between the groups were not significant (*p* = 0.18).

## Discussion

Recent reviews and meta-analyses have reported that use of vaginal pessaries does not cause deterioration of sexual function in POP patients. In contrast, some evidence has indicated that pessary use improves sexual function [6, 15, 21–23]. In the present study, we found that long-term use of vaginal cube pessaries had a positive effect on the sexually active POP patients.

Our secondary analysis of a prospective cohort study examined the long-term sexual experiences of symptomatic POP patients treated with cube pessary self-management. We found that 68% of all sexually active patients described their sexual lives as “better” or “much better” than before the treatment. No patient reported deterioration in their sexual life. Our findings demonstrate that the sexual wellbeing and QoL of POP patients can be significantly improved in the long term. The sexually active patients had a better QoL, as measured by the PGI-I, than the sexually inactive patients. Of the sexually active patients, 91% reported their condition to be much better or better than before treatment.

According to current medical views, a correctly fitted support pessary should not cause any harm or discomfort during sexual intercourse; however, the majority of patients and their partners are bothered by the presence of the pessary. Meriwether and colleagues reported that 70% of sexually active patients “usually” or “always” removed their pessary before sexual intercourse [24]. The reasons most commonly cited were that the “partner can feel it during sex” (31%) and that the “pessary is uncomfortable during sex” (20%) [24]. These negative influences on sexual function can be easily managed by removing the pessary before intercourse. Cube pessaries allow sexual intercourse to be achieved without having the pessary in situ. In a previous study of 43 women conducted after 1 year of pessary self-care, we found that 20 of the sexually active patients (46.5%) reported an improvement in their sex life following pessary use, whereas 23 (53.5%) reported no change [19]. A common criticism of vaginal cube pessaries is that having to remove them prior to sexual activity is a “mood killer.” However, the vast majority of our sexually active patients (91.2%) reported that pessary removal was not disruptive and did not cause inconvenience during the 5-year study period. All but one of the participants described the self-fitting procedure as easy or very easy in our primary analysis of this study [16].

Physicians have commonly considered sexual activity to be a relative contraindication to long-term pessary treatment [25]. However, our study results and prior research suggest that sexual activity promotes the long-term use of pessaries [26]. Another important factor when considering long-term pessary treatment is the possibility of self-management. In an analysis of predictors for continued pessary use, self-care was the only factor that influenced compliance rates after treatment for 3 years [27]. Inability to carry out self-care was associated with discontinuation of pessary use, according to Manchana and colleagues [28].

A recent systematic review and meta-analysis suggested that the main risk factors for prolapse recurrence after native tissue surgery might be preoperative prolapse stage and a younger age [29]. Therefore, pessary self-management should be offered as a first-line therapy for younger, sexually active women with symptomatic POP to improve their QoL [16].

One limitation of this study is that we only studied patients using cube pessaries made by Arabin® (Witten, Germany). It is unclear if the use of pessaries produced by other manufacturers would have the same results. Another limitation is that sexually active postmenopausal patients also used local estrogen therapy during the study. The positive results experienced by sexually active postmenopausal patients may be partially attributed to this therapy. Some participants answered our questions in privacy online, whereas others responded over the phone, but as the collection of the data was carried out in multiple locations, a comparison of these two subgroups is lacking. Finally, we could not assess detailed sexual functions, as we did not use translated and validated questionnaires.

## Conclusion

Daily self-management of cube pessaries allows continuous sexual activity, which has a positive impact on an individual’s quality of life, body image, and relationship. Our results demonstrate that the majority of sexually active long-term vaginal pessary users experienced improvement in their sexual lives, and most individuals experienced no problems in removing the pessary before sexual activity.

## Data Availability

Data analysed is available upen request from the first author.
